# Total thoracoscopic lung segmentectomy of anterior basal segment of the right lower lobe (RS8) for NSCLC stage IA (case report)

**DOI:** 10.1186/1749-8090-6-115

**Published:** 2011-09-24

**Authors:** Masahiro Miyajima, Atsushi Watanabe, Mayuko Uehara, Takuro Obama, Junji Nakazawa, Tomohiro Nakajiima, Keishi Ogura, Tetsuya Higami

**Affiliations:** 1 Department of Thoracic and Cardiovascular Surgery, Sapporo Medical University Chuo-ku, S1W16, Sapporo, Hokkaido0608543, Japan

## Abstract

A 69-year-old woman with a pulmonary nodule in anterior basal segment of the right lower lobe (RS8) was referred to our department. The diameter of the tumor was 12 mm, and it had increased over a few months. First, video-assisted thoracoscopic lung surgery (VATS) biopsy of the pulmonary nodule was carried out. Frozen section examination of this nodule confirmed the diagnosis of bronchioloalveolar carcinoma (BAC). Segmentectomy of RS8 with lower mediastinal node dissection (ND2a-1) was performed. The intersegmental plane was identified using the intersegmental veins as landmarks and the demarcation between the resected (inflated) and preserved (collapsed) lungs. Electrocautery at 70 watts was used to divide the intersegmental plane. A vessel sealing system was used to seal and cut the pulmonary arteries. Postoperative histopathological examination revealed that the tumor was T1aN0M0 BAC, and the minimal distance between the surgical margin and the tumor edge was 15 mm. The patient was discharged from hospital on postoperative day 5 without any complications.

## Background

Segmentectomy for non small cell lung cancer (NSCLC) stage I patients still remains controversial. The late outcomes of lung function and underlying diseases are unclear. We are currently looking forward to the results of the two ongoing randomized, controlled studies: a study conducted by the Cancer and Leukemia Group B (CALGB14053), a phase III randomized trial of lobectomy versus sublobar resection for small (< 2 cm) NSCLC; and a similar phase III randomized study conducted by the Japan Clinical Oncology Group (JCOG) and the West Japan Oncology Group (WJOG) (JCOG0802/WJOG4607L) [1]. There are few reports on VATS segmentectomy [2,3]. The procedure has some drawbacks: it is technically demanding, and it is difficult to comprehend the anatomical relations among the bronchus, pulmonary arteries and pulmonary veins. Preoperative three-dimensional contrast-enhanced computed tomography (3D-CT) simulation and the use of a vessel sealing system (VSS) to cut the vessels and dissect the parenchyma make this complicated surgery easier and more practical [4,5]. The case of a patient with VATS anterior basal segment of the right lower lobe (RS8) segmentectomy for stage IA NSCLC is presented.

## Case Presentation

A 69-year-old woman who was diagnosed with a lung tumor was admitted to our hospital. The greatest diameter of the tumor was 12 mm, and it had increased over several months. Bronchoscopy did not yield a definitive diagnosis, so Thoracoscopic surgery for diagnosis and treatment was scheduled. Past medical history included mitral valve insufficiency treated with oral medication. Family history was unremarkable, and she had never been a smoker. The physical examination was normal. The results of the laboratory investigations, including a complete blood count, liver and renal function tests, coagulation studies and the serum cancer antigens, were within the normal range.

**Figure 1:**
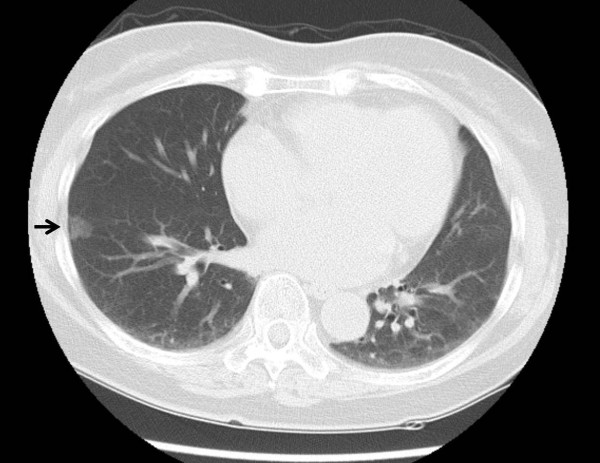
**Chest CT revealed a tumor (arrow) in anterior basal segment of the right lower lobe (RS8)**.

**Figure 2:**
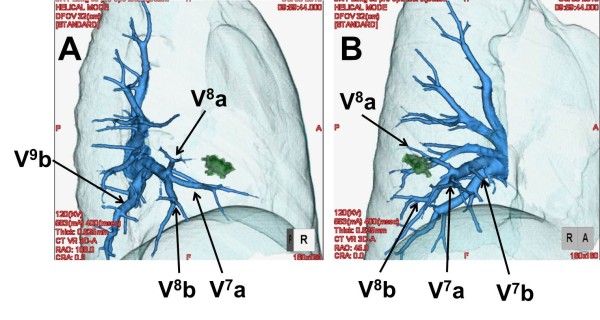
**Images of the right lower pulmonary veins**. On this image, the relationships of the intersegmental veins (V^8^a and V^8^b) that demark anterior basal segment (S8) and lateral basal segment (S9) and the veins (V^7 ^and V^9^b) that should be preserved can be clearly demarcated.

**Figure 3:**
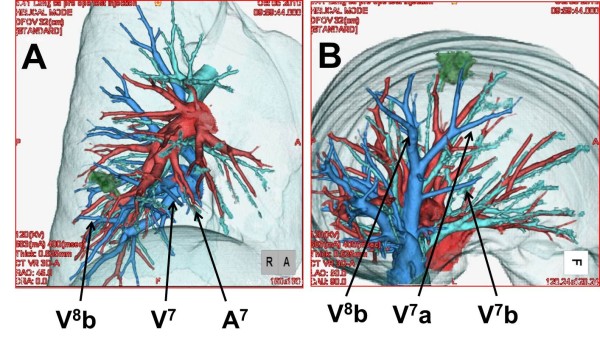
**Images from three-dimensional computed tomographic angiography of the right lower lobe of the lung**. The complicated anatomy of the pulmonary arteries (red), pulmonary veins (blue), and bronchus (green) is precisely depicted. From this image, the intersegmental plane between medial basal segment (S7) and anterior basal segment (S8) can be easily imagined.

Pulmonary function tests showed that vital capacity (VC) was 2590 ml, percentage of predicted VC was 109.3%, forced expiratory volume in 1 s (FEV1) was 2120 ml and FEV percentage in 1 s was 81.9%. Chest computed tomography demonstrated a partilally serrated border 12-mm-diameter pulmonary nodule in anterior basal segment of the right lower lobe (RS8)(Figure 1). To guide the surgeons in simulating the operation, preoperative three-dimensional (3D)-CT was performed. Using 3D volume rendering, a solid image was constructed from 0.65-mm data slices of the contrast-enhanced CT images. A colored map was used to highlight the blood vessels of the lung. The 3D rendered images were magnified, de-magnified, and rotated to examine these measurements (Figures 2, 3). To secure an adequate margin from the tumor, preoperative needle marking was performed under CT guidance on the day before surgery. The needle marker (Guiding Marker System; Hakko Medical Products, Tokyo, Japan) was put around the tumor [6].

**Figure 4:**
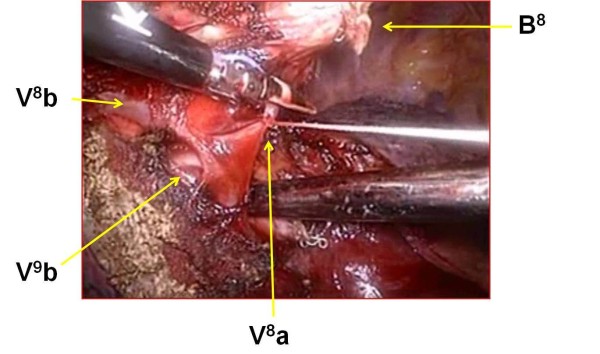
**Operative view**. On this view, the intersegmental veins (V^8^a and V^8^b) that demark anterior basal segmet (S8) and lateral basal segment (S9) can be seen to have been resected with the VSS. The intersegmental vein (V^9^b) that should be preserved is well identified.

VATS needle biopsy was then planned, with subsequent total thoracoscopic segmentectomy of anterior basal segment of the right lower lobe (RS8) if the diagnosis was malignancy. Our indication criteria for segmentectomy are clinical T1aN0M0 peripheral NSCLC. The segments for resection are determined based on tumor size and peripheral location in order to critically secure a segmental margin free of tumor cells. Segmentectomy is converted to lobectomy when the intraoperative node sampling shows node involvement. Under general anesthesia with single lung ventilation and thoracic epidural anesthesia, the patient was placed in the left decubitus position. The surgeon was positioned on the anterior side of the patient. Two thoracoports were placed in the sixth intercostal space (ICS) at the anterior axillary line and the seventh ICS at the posterior axillary line. The anterolateral 30-mm access port was placed in the fourth ICS. A Lap Protector Mini (Hakko Medical Co., Tokyo, Japan) was placed on the site of the access port. A 30-degree scope was used. The interlobular fissure was almost complete. Frozen section examination of the needle biopsy specimen confirmed the diagnosis of BAC, and VATS segmentectomy of RS8 with node dissection was performed. First, the intermediate pulmonary artery, the middle lobe pulmonary artery, A6, A7, and A8 were exposed in the interlobar site. Proximal A8 was ligated with 2-0 silk, and distal A8 was sealed and then divided with the VSS. The intersegmental plane was identified using the intersegmental veins as landmarks and the demarcation between the resected (inflated) and preserved (collapsed) lungs. This status of the lung was induced by the following three steps: temporarily re-inflating the whole lung, ligating the resected segmental bronchus (B8), and deflating preserved lung. After making the demarcation, ligated B8 was then stapled and divided with an endoscopic stapler. The intersegmental plane of the parenchyma was divided by electrocautery by 70 Watts from the pleural surface of the lung[7,8]. VSS was also used to dissect along the intersegmental veins. The intersegmental veins (V^8^a and V^8^b) that demark S8 and lateral basal segment (S9) were resected with RS8 after sealing and division with the VSS, while the intersegmental vein (V7) that demarks medial basal segment (S7) and S8 was preserved (Figure 4). Finally, air leaks were repaired with several 4-0 PDS horizontal mattress sutures with absorbable pledgets (Medifit Felt, JMS Co., Hiroshima, Japan). Absorbable polyglycol acid sheets (NEOVEIL^® ^sheet, Gunze, Ayabe, Japan) were applied to the intersegmental plane with fibrin glue to obviate further air leaks.

The operative time was 221 min, and blood loss was 30 ml. The chest drainage tube was removed on postoperative day 2. The patient was discharged from hospital on postoperative day 5 without any complications. Postoperative histopathological examination revealed that the tumor was T1aN0M0 BAC. The minimal distance between the surgical margin and the tumor edge was 15 mm, and the margin was free of malignancy.

## Discussion

Along with the recent development of radiographic devices such as high-resolution computed tomography and the widespread practice of low-dose helical computed tomography for screening, early detection of ever-smaller NSCLS has increased.

It remains to be established whether segmentectomy is an appropriate procedure for NSCLC patients who can tolerate lobectomy. The potential advantage of segmentectomy compared with lobectomy is the preservation of pulmonary function, whereas in comparison with wedge resection, an improved oncologic outcome is noted with segmentectomy [9]. This concept may be addressed by a study conducted by the Cancer and Leukemia Group B (CALGB14053), a phase III randomized trial of lobectomy versus sublobar resection for small (< 2 cm) NSCLC.

Our indication criteria for segmentectomy are clinical T1aN0M0 peripheral NSCLC. The segments for resection are determined based on tumor size and peripheral location in order to critically secure a segmental margin free of tumor cells. The segmental, lobar and mediastinal nodes should be carefully sampled and confirmed to be cancer negative by frozen section examination. Segmentectomy is converted to lobectomy when the intraoperative node sampling shows node involvement.

Reports on thoracoscopic segmentectomy were limited to segments that can be easily excised. In case of such segments, it is possible to simultaneously separate the lung parenchyma from both the hilum and the periphery by using staplers. Segmentectomy used to be difficult for the other segments. In such segments, digital dissection of the segments had been necessary. Owing to the recent development of the pre-operative 3D-CT and the improved quality and resolution of scans thus obtained, total thoracoscopic lung segmentectomy of these segments have been reported[4,8].

In the present case, as a result of the pre-operative 3D-CT simulation, we could comprehend the precise anatomy of the complicated vessels and the bronchi. Especially, it is important to identify the intersegmental veins for total thoracoscopic lung segmentectomy[4].

In the limited working space, usage of the VSS was very safe and useful to expose and divide pulmonary vessels. During a total thoracoscopic lung segmentectomy, suture ligation of PA and the treatment of intraoperative bleeding can be more challenging than during segmentectomy by a video-assisted mini-thoracotomy. It is assumed that energy-based vessel sealing and cutting instruments reduced difficulties in dividing pulmonary blood vessels in total thoracoscopic lung segmentectomy. Compared to VSS, the ultrasonic device consistently generates higher temperatures (200°C vs 94.3°C). It has been reported that compared to the VSS, the ultrasonic device required twice as long to cool off, and the mean Burst Pressure was lower[10]. Therefore, for use in limited thoracic space, and for sealing and cutting of pulmonary blood vessels, VSS is more suitable.

Once the intersegmental plane has been determined, the last issue is the choice of the segmental division method. Some including us use a combination of electrocautery and application of fibrin sealant[7,8]. But most use staplers[2,3,9]. The application of stapling can often compromise adjacent pulmonary parenchyma, restricting full expansion of the residual segments and thus pulmonary function. On the other hand, post operative air leakage was the major problem when using an electric cautery. In patients with severe emphysematous changes, stapling device may be applied for stringent control of air leaks.

We believe that this technique will contribute to improved outcomes in selected lung cancer patients.

## Conclusion

With the help of pre-operative 3D-CT simulation of the complicated vessels and the bronchi, as well as VSS to expose and divide pulmonary vessels, total thoracoscopic segmentectomy of anterior basal segment of the right lower lobe (RS8) was safely performed.

## Competing interests

The authors declare that they have no competing interests.

## Authors' contributions

MM conceived of the study, drafted the manuscript, and participated in its design and coordination.

AW conceived of the study, and participated in its design and coordination.

MU participated in this surgical operation and took care of the patient.

TO participated in this surgical operation and took care of the patient.

JN participated in this surgical operation and took care of the patient.

TN participated in this surgical operation and took care of the patient.

KO carried out the pre-operative 3D-CT imaging.

TH participated in its design and coordination.

All authors read and approved the final manuscript.
